# A mathematical modelling tool for predicting survival of individual patients following resection of glioblastoma: a proof of principle

**DOI:** 10.1038/sj.bjc.6604125

**Published:** 2007-12-04

**Authors:** K R Swanson, R C Rostomily, E C Alvord

**Affiliations:** 1 Laboratory of Neuropathology, Department of Pathology, University of Washington, Seattle, WA, USA; 2 Department of Applied Mathematics, University of Washington, Seattle, WA, USA; 3 Department of Neurological Surgery, University of Washington, Seattle, WA, USA

**Keywords:** glioblastoma, invasion, MRI, mathematical model, proliferation, resection

## Abstract

The prediction of the outcome of individual patients with glioblastoma would be of great significance for monitoring responses to therapy. We hypothesise that, although a large number of genetic-metabolic abnormalities occur upstream, there are two ‘final common pathways’ dominating glioblastoma growth – net rates of proliferation (*ρ*) and dispersal (*D*). These rates can be estimated from features of pretreatment MR images and can be applied in a mathematical model to predict tumour growth, impact of extent of tumour resection and patient survival. Only the pre-operative gadolinium-enhanced T1-weighted (T1-Gd) and T2-weighted (T2) volume data from 70 patients with previously untreated glioblastoma were used to derive a ratio *D*/*ρ* for each patient. We developed a ‘virtual control’ for each patient with the same size tumour at the time of diagnosis, the same ratio of net invasion to proliferation (*D*/*ρ*) and the same extent of resection. The median durations of survival and the shapes of the survival curves of actual and ‘virtual’ patients subjected to biopsy or subtotal resection (STR) superimpose exactly. For those actually receiving gross total resection (GTR), as shown by post-operative CT, the actual survival curve lies between the ‘virtual’ results predicted for 100 and 125% resection of the T1-Gd volume. The concordance between predicted (virtual) and actual survivals suggests that the mathematical model is realistic enough to allow precise definition of the effectiveness of individualised treatments and their site(s) of action on proliferation (*ρ*) and/or dispersal (*D*) of the tumour cells without knowledge of any other clinical or pathological information.

Gliomas are well known as extensively invasive lesions with a variety of genetic-metabolic abnormalities that contribute to their uncontrolled proliferation and invasion. A mathematical model has been developed based on the two final common paths of these mechanisms – proliferation and invasion. Serial medical imaging can be used to track the spatio-temporal behaviour of the detectable portion of each lesion, but the undetectable and undefinable fraction of the neoplasm remains as a problem.

The original mathematical model (Tracqui *et al*, 1995), based on the analysis of a single patient followed with serial CTs during his final year, provided values of rates of net proliferation (*ρ*) and net diffusion (*D*) that were used as averages for populations of hypothetical patients (Woodward *et al*, 1996) to compare with populations of real patients (Kreth *et al*, 1993) subdivided only by extent of surgical resection, biopsy or ‘gross total resection’ (GTR). A difference in median survival of 7 weeks was predicted mathematically, and actually found, although not statistically significant, even with 115 patients (Kreth *et al*, 1993). Other modelling efforts in the area of glioma have focused either on multicellular spheroids *in vitro* (Stein *et al*, 2007) or on the averages of groups of patients (Morris *et al*, 2006). Our mathematical modelling approach has increasingly focused on the individual patient, as in this study.

The current study is motivated by several other recent applications of our modelling technique. Specifically, our discovery that the model-predicted linear radial growth of the imaging-detectable portion of the lesion (at a velocity dependent on both *ρ* and *D*) was accurate in a population of WHO grade II gliomas followed serially without intervening treatment (Mandonnet *et al*, 2003) which led to confirmation that the velocity of growth predicts survival time in a similar population (Pallud *et al*, 2006). However, since higher grade gliomas (glioblastomas) are typically treated immediately, a similar population of serially imaged untreated glioblastomas has been difficult to obtain and only a few cases have been reported (Swanson and Alvord 2002; Harpold *et al*, 2007). Here we demonstrate how our mathematical model, adapted to accommodate a level of resolution of MRIs, from which could be derived a ratio of *D*/*ρ* for any glioblastoma patient (Swanson, 1999; Swanson and Alvord 2002; Swanson *et al*, 2002; Alvord *et al*, 2003; Swanson *et al*, 2003a, 2003b), can be used to help estimate the undetectable, diffusely invading component of gliomas and to predict survival of individual patients.

It should be emphasised that the model uses only currently available diagnostic pre-operative MR imaging characteristics of individual patients, the only necessary requirement being the presence of contrast enhancement, and uses no other clinical or statistical interpretation, not even the histologic diagnosis of glioblastoma (although all of the present patients have such a diagnosis). It should also be emphasised that model parameters *D* and *ρ* are net, downstream from any intracellular metabolic activity controlling migration and proliferation, irrespective also of any microenvironmental factors that might further modify them. In contrast to the original comparison of virtual and real patients (Woodward *et al*, 1996), where individual sizes were not known and where averages from the scanty literature were available at the time of diagnosis (Blankenberg *et al*, 1995) and at death (Concannon *et al*, 1960; Burger *et al*, 1988) had to be used, the present series was able to use not only the individual sizes known for each patient at diagnosis, but also the confirmation of the completeness of resections by post-operative enhanced CT, not just the surgeon's opinion (Kreth *et al*, 1993). The results suggest the potential utility of such modelling (avoiding all statistically defined qualifiers, including age, Karnofsky Performance Score (KPS), histological type and grade, etc.) in future clinical studies comparing specific biomarkers and treatments of gliomas in individual patients.

## MATERIALS AND METHODS

### The patients

The 70 patients were adults (22–79 years old, mean age 58 years) with supra-tentorial glioblastoma diagnosed, treated and followed at the University of Washington Medical Center in 1993–1995. Follow-up was complete to death in 66 patients and to 104, 208, 230 and 230 weeks in the 4 patients who were lost to follow-up. All of the patients received X-irradiation to the tumour of 59.4 Gy or more, 58 received chemotherapy and 30 s operations. Our Institutional Review Board approved this retrospective study of patients.

**Figure 1:**
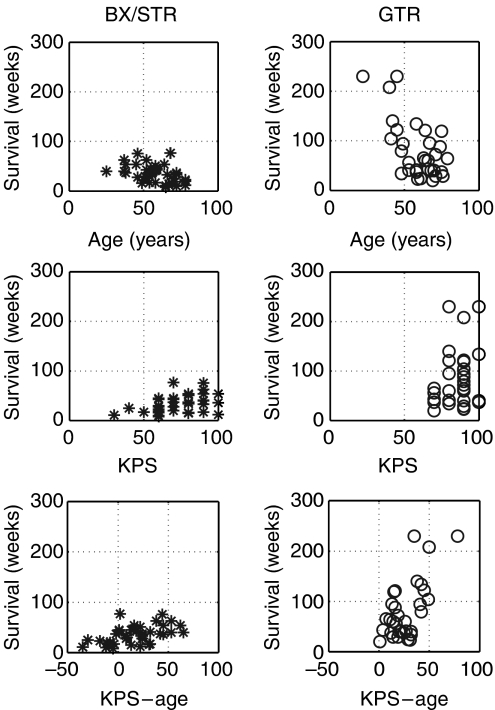
Scatter graphs defining some of the major characteristics of the 70 patients with correlations of duration of survival for BX/STR patients (asterisks) and for the GTR patients (squares) with age, KPS and KPS-age. Note the broad ranges, illustrating the variety of the patients. Note also that none of these data enter into the mathematical model.

Of the 70 patients, 58 had lobar tumours and 12 had tumours in deep or mixed location; 58 had tumours that anatomically made them eligible for ‘gross total resection’ (GTR), but 20 of these were reclassified as only ‘subtotal resection’ (STR) when post-operative enhanced CT revealed a nodule of residual tumour. Of the 18 whose tumours were too extensive even to consider GTR, 11 had STR and 7 only biopsy. Major clinical characteristics are summarised in Figure 1, which shows the durations of survival following the specific treatments correlated (*P*<0.01 Student's *t*-test) with age and KPS and demonstrates the broad ranges of clinical factors that can be accommodated by – but not incorporated in – the mathematical model.

The diagnosis of glioblastoma was generally the result of a consensus of four neuropathologists at a time when one of the present authors (ECA) was chief of neuropathology and required necrosis (Nelson *et al*, 1983) as one of the histologic criteria of glioblastomas. No distinction was made between various subtypes, including primary or secondary, as later suggested by Kleihues *et al* (2002).

**Figure 2:**
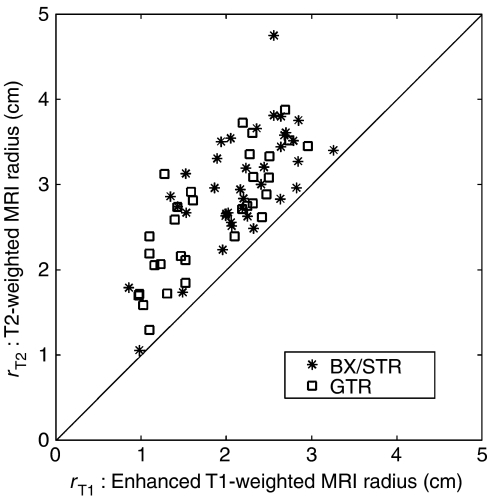
The actual data used for the present analysis: average radii of spheres equivalent to the T1-Gd and T2-weighted MRI volumes for glioblastomas of 70 actual patients subjected to biopsy or subtotal resection (BX/STR, asterisks, *N*=38), or to gross total resection (GTR, squares, *N*=32) as defined by the absence of tumour on post-operative enhanced CT.

We used the pre-operative tumour volumes as defined by gadolinium-enhanced T1-weighted (T1-Gd) and T2-weighted (T2) MR imaging. These volumes had been digitised and calculated using NIH Image software that added the areas on each slice and multiplied the sum by the thickness of each section to obtain the volume. Two independent observers had made the measurements and the mean was accepted. The percent differences between the two observers was 7.6% (±7.3%) and 6.0% (±4.7%) for T1-Gd and T2 measurements, respectively. The total volumes included any central necrosis. From these volumes we calculated the radii of equivalent spheres (Figure 2), and these radii were the only data entered into the mathematical formulations.

We used other clinical data only to define appropriate subsets to be compared as to durations of survival, actual *vs* predicted (virtual). These data included statements as to whether the patient had received steroids, what type of surgery had been performed, and the duration of survival from the date of operation. The extents of resection included only statements as to biopsy only, subtotal and gross total resection, the latter two arbitrarily defined by the presence or absence of residual tumour on post-operative contrast-enhanced CT, performed within 2 days following surgery.

### Model development

The mathematical model continues to be based purely on the classical definition of cancer as uncontrolled proliferation of cells with the potential for invasion and metastasis, simplified for gliomas, which practically do not metastasise. Thus, the model defines the behaviour of gliomas in words and mathematics as follows:
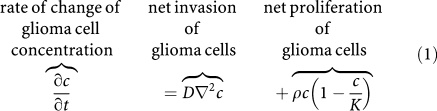


This is a classical conservation–diffusion equation (Murray, 2003), in which *c*(**x**, *t*) defines the concentration of malignant cells at location **x** and time *t*, *D* (mm^2^ day^−1^) is the random motility (dispersal) coefficient defining the net rate of migration of the tumour cells, *ρ* (per day) represents the net proliferation rate of the tumour cells (including mitosis and cell loss), *K* is the limiting concentration of cells that a volume of tissue can hold (i.e., the carrying capacity of the tissue) and ∇^2^ represents the dispersal operator, the Laplacian, expressed mathematically as the sum of three second derivatives in space (Strang, 1991). The model has been adapted to use the BrainWeb Atlas (Collins *et al*, 1998) to accommodate an irregularly shaped tumour located anywhere within 3-dimensionally continuous heterogeneous tissue with differences in grey and white matter, anatomically accurate to 1 mm^3^ (Swanson, 1999; Swanson *et al*, 2003a). The model can accommodate different velocities of glioma cell motility in grey and white matter (Swanson, 1999) but, since the original MRIs were not available, this feature was not used in the present analysis.

**Figure 3:**
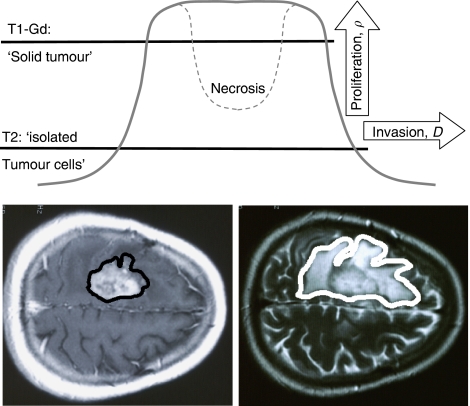
Schematic representations of the relationships between T1-Gd and T2 images and the concentrations of tumour cells and of the effects of increasing the net rates of diffusion (*D*) and proliferation (*ρ*) on the ‘traveling wave’ of the ‘edge’ of an enlarging glioblastoma. The thresholds of detection for T1-Gd and T2 MR images (solid lines) are estimated at 80 and 16%, respectively, of the maximum cell density *K* in Equation (1).

Equation (1) implies mathematically that the ‘edge’ of the visible tumour advances asymptotically as a ‘traveling wave,’ (Swanson, 1999) which expands radially and linearly, according to Fisher's approximation or Skellam's model (Shigesada and Kawasaki, 1997): 
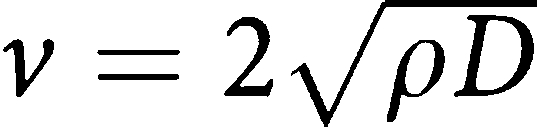
. Although there is no true edge to an infiltrating tumour, such as a glioma, any point on the ‘gradient’ between the T1-Gd and T2 circumferences (Figure 3) moves as part of the ‘traveling wave.’ In general, as shown schematically in Figure 3, proliferation (*ρ*) tends to drive the wave up (but not above the carrying capacity *K*) and dispersal (*D*) tends to drive the gradient centrifugally.

The ‘gradient’ between the T1-Gd and T2 images can be expressed in a different way, involving the ratio *D*/*ρ*. This ‘gradient’ has not been quantitatively defined but can be approximated from the observations of Kelly *et al* (1987), Kelly (1993) and Dalrymple *et al* (1994), who reported that the T1-Gd circumference approximates the edge of the ‘solid tumour’ and that the T2 circumference represents not only the extent of oedema but also a zone of a low concentration of ‘isolated tumour cells.’ We hypothesised that these circumferences might represent concentrations of tumour cells equal to 80 and 16%, respectively, of the maximum concentration (Figure 3). That tumour cells extend much farther then even the imageable abnormality is evidenced by malignant cells being cultured by Silbergeld and Chicoine (1997) from as far away as 4 cm. A close study of other solutions of the model Eq. (1) finds a highly non-linear relationship between the ratio *D*/*ρ* and the average radii of spheres equivalent to the volumes defined by T1-Gd and T2, *r*_T1_ and *r*_T2,_ respectively (Harpold *et al*, 2007). The equation by no means is a simple ratio of any part(s) of the MR images, but includes fractional exponents of ratios of differences that make it highly non-linear.

Our model consists of the following two parts: (1) Eq. 1, the spatio-temporal bio-mathematical formulation of the proliferation and dispersal of the tumour cells, both visible (detectable by scans) and invisible (diffusing into the surrounding normal-appearing tissue), and (2) the use of Fisher's approximation (
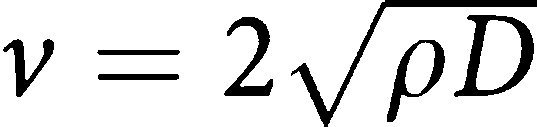
) to estimate the time required for the tumour to expand from its detectable actual size at diagnosis to its size at death (Woodward *et al*, 1996).

### Data analysis

Statistical analysis was performed (SPLUS, 1998), as noted throughout the text. From the pre-operative MRI volumes, we calculated the average radii of equivalent spheres of the T1-Gd and T2 volumes for each of the 70 patients (Figure 2). These became the actual data from which all of the rest of the calculations derive (see Results section). The mean T1-Gd radius was 2.01 cm, the median 2.13 cm and the range from 0.86 to 3.26 cm. The mean T2-weighted radius was 2.89 cm, the median 2.84 cm, and the range from 1.05 to 4.75 cm.

We used other clinical data provided to classify each actual patient according to the extent of resection. We found that there is no statistically significant difference between the means of the T1-Gd radii of the 7 biopsy patients (2.03 cm) and the 31 STR patients (2.20 cm, *P*=0.43) or of the 7 biopsy and 32 GTR patients (1.81 cm, *P*=0.44), in large part because of the small number of biopsy patients (*N*=7); but there is a significant difference between the means of the T1-Gd radii of the combined 38 biopsy and STR patients (mean=2.17 cm) and the 32 GTR patients (*P*=0.01 using Student's *t*-test). Justification for this combination of patients is provided in the Results section.

**Figure 4:**
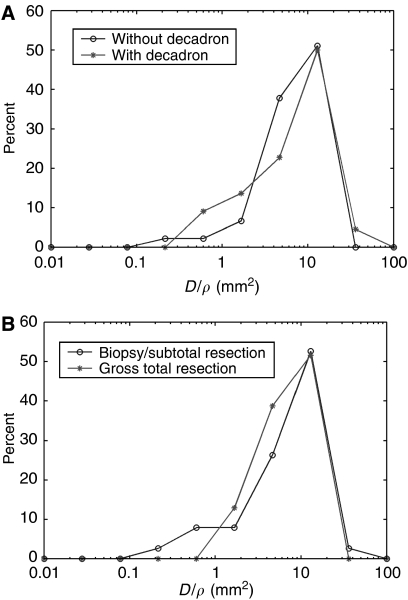
Distributions (%) of actual values of *D*/*ρ*, in (**A**) for 22 patients having had steroid (asterisks) and for 45 patients not having had steroid (circles) before their MRIs, and in (**B**) for 38 patients having had biopsy or subtotal resection (circles) and 32 patients having had gross total resection (asterisks), as defined by the presence or absence of residual tumour in post-operative enhanced CT.

From the diagnostic MRI tumour volumes, we have also calculated the actual ratio, *D*/*ρ*, for each of the 70 patients and found the median to be 9.83 mm^2^, the mean 10.52 mm^2^ and the range from 0.24 to 35.92 mm^2^. There is no significant difference in the distributions of the ratio *D*/*ρ* between actual patients who had had steroids for a few days (*N*=22) and those who had not had steroid (*N*=45) before their MRIs (Figure 4A, *P*=0.75 by Kolmogorov-Smirnov's test (K-S test)), Watling *et al*, (1994) having used steroids for a longer time to see any effect. There is also no difference in the distributions of the ratio *D*/*ρ* for the actual biopsy, STR and GTR patients (Figure 4B, K-S test *P*=0.675).

In the absence of a second set of MRIs to define the actual velocity for each patient, we had to resort to estimates, applying the average *ρ* of 0.012 per day (Tracqui *et al*, 1995; Woodward *et al*, 1996) to the calculated actual ratios of *D*/*ρ* to estimate *D* for each actual patient. We could then apply Fisher's approximation (
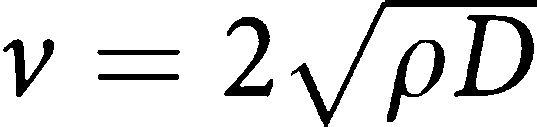
) to calculate an approximate radial velocity of expansion of the ‘edge’ of each tumour. We found the median velocity to be 0.075mm day^−1^, the mean to be 0.0723 mm day^−1^, and the range from 0.0118 to 0.1438 mm day^−1^. Since the ratio of *D*/*ρ* is known from the MRIs of each patient, any different value of *ρ* would induce a corresponding change not only in this estimated value of *D* but also in the velocity *v*. We accepted the average size at death as equivalent to a sphere of radius 3 cm (Woodward *et al*, 1996) and allowed the model to project each tumour at its estimated velocity *v* and to determine the time required to grow from its actual diagnostic T1-Gd radius to the assumed fatal radius of 3 cm. These times were the predicted virtual durations of survival, which could be calculated not only without treatment but also by superimposing virtual resections of any extent desired. Any or all of these predicted survival times could be compared with the appropriately matched actual durations of survival (see Results section).

Estimates of the predicted survival time for each of the 70 patients were made following a variety of ‘virtual’ treatments: (1) biopsy only (no resection) and STR (treated as no resection, as in Woodward *et al*, 1996), (2) resection of 100% of the T1-Gd volume (the smallest ‘virtual GTR’ that could have accomplished the CT-proven result) and (3) resection of 125% of the T1-Gd volume (an arbitrarily chosen estimate of a more extensive ‘virtual GTR’ that the surgeon could have easily undertaken). Groups 2 and 3 were predicted to develop a range of predictions for comparison with the actual GTR patients, in this data set defined by the absence of residual tumour on post-operative enhanced CT. It seems obvious that the exact extent of resection beyond complete removal of the CT-enhancing tumour was not determined in any actual patient and was not likely to have been the same in each patient. Virtual resection was simulated, as by Swanson *et al* (2003a), by imposing a cell concentration of zero in the resected region and allowing the computer to proceed with the model-predicted tumour growth defined by Eq. (1).

## RESULTS

**Figure 5:**
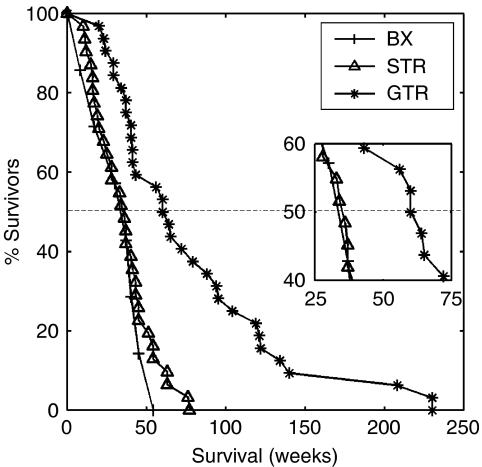
Actual survival curves for 7 patients subjected to biopsy (BX, pluses), for 31 subjected to subtotal resection (STR, triangles) and for 32 subjected to gross total resection (GTR, asterisks) as defined by presence or absence of residual tumour on post-operative enhanced CT. Inset shows a close-up of the survival curves near the median survival times of 36.5 and 62 weeks, respectively.

**Figure 6:**
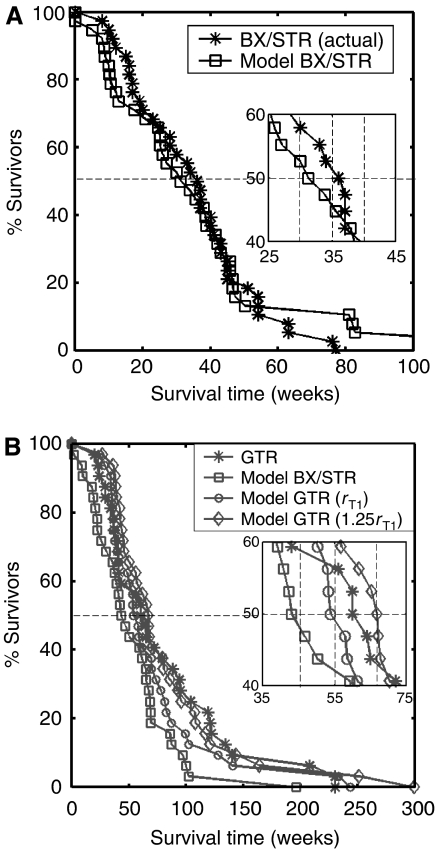
(**A**) Survival curves for actual patients (asterisks) and virtual patients (squares) subjected to biopsy or subtotal resection (BX/STR, *N*=38). Inset shows a close-up of the survival curves near the median survival times of 32.4 and 36.5 weeks. (**B**) Survival curves on a longer time scaling following gross total resection (GTR, *N*=32) in actual patients (asterisks) defined by the absence of residual tumour on post-operative enhanced CT. The virtual patients (matched to actual pre-operative T1-Gd volume and *D*/*ρ* ratio derived from the T1-Gd and T2 volumes) were subjected to no resection (BX/STR, squares), to resection of 100% of the T1-Gd volumes or radii, *r*_T1_ (circles) and to resection of 125% of the T1-Gd volumes or radii, 1.25 *r*_T1_ (diamonds). Inset shows a close-up of the survival curves near the median survival times of 44.9, 55, 62 and 66.9 weeks.

We begin by presenting Figure 5, summarising the actual durations of survival for the 7 patients subjected to biopsy only, the 31 patients subjected to STR and the 32 patients subjected to GTR as defined by post-operative enhanced CT. Since there were so few biopsy patients and since there was no difference in actual durations of survival compared with the STR patients (*P*=0.671, *χ*^2^-test), consistent with previous studies of patients receiving Bx or STR (Lacroix *et al*, 2001) these two actual groups were combined in further comparisons. These actual results (Figure 5) are meaningless until matched with appropriate controls (see Figure 6 below).

In Figure 6, we have paired the virtual and real patients for appropriate comparisons. It should be noted that these comparisons are being made between real and virtual patients with exactly the same pre-operative MRI characteristics defined by the actual size (T1-Gd volume) and the actual ratio *D*/*ρ* (derived non-linearly from the actual T1-Gd and T2 volumes). The actual survival curves are represented as asterisks and the virtual survival curves as open boxes of various shapes corresponding to the various extents of resection. Although the degree of overlap represents the degree of accuracy of prediction, enlargements are provided in the insets to reveal the slight discrepancies present. In Figure 6A, the untreated virtual patients are compared with the actual patients subjected to biopsy and STR. The curves are practically identical (no significant difference *P*=0.693, *χ*^2^-test), as predicted by Woodward *et al* (1996) and as found by Lacroix *et al* (2001). In Figure 6B, the untreated and treated virtual (100 and 125% resections, respectively) and actual patients subjected to GTR are compared. As expected, the actual curve lies between the 100 and 125% ‘virtual’ resections. Neither of the virtual curves was significantly different from the actual (*P*=0.695, 0.685, respectively, *χ*^2^-test).

## DISCUSSION

The real power of any model is its ability to provide meaningful predictions (Murray, 2003), as seen most graphically in Figure 6. The present mathematical model, although requiring a computer for solutions, is relatively simple and based biologically on the classical definition of cancer/gliomas. It reduces the significant variables to only two, the ‘final common paths’ of net proliferation (*ρ*) and dispersal (*D*) rates. All of the genetic-metabolic abnormalities that are being revealed to underlie the mysteries of glioblastomas lie upstream but their downstream net effects can be quantified by the model. The model has allowed us to provide almost exact virtual controls, matched for pre-operative size (T1-Gd) and *D*/*ρ*, untreated and treated, for each patient, and to make accurate predictions of survival times following a broad range of surgical resections, as shown in Figure 6. As Woodward *et al* (1996) predicted mathematically and as shown actually in Figure 5, there is no difference in duration of survival between real patients receiving biopsy only and those receiving STR (*P*=0.671, *χ*^2^-test). As for the evaluation of the effectiveness of GTR, a subject of almost limitless controversy (Nazzaro and Neuwelt, 1990; Kelly, 1993; Kreth *et al*, 1993, 1999; Kowalczuk *et al*, 1997; Hess, 1999; Keles *et al*, 1999; Lacroix *et al*, 2001), we would emphasise Figure 6, where the virtual ‘untreated’ controls (asterisks) for each subset (biopsy/STR and GTR) differ in duration of survival (32.4 *vs* 44.9 weeks). There being no difference in the distributions of the actual ratios *D*/*ρ* (Figure 4B), the only remaining reason for these differences in survival is that the individually matched T1-Gd sizes differ for the two subsets, as can also be seen in Figure 2. Both Figure 2 and Figure 6 suggest that the ‘better’ patients were chosen for GTR.

Thus, comparisons between the real (unmatched) subsets (Figure 5) are, in our opinion, not so appropriate as comparisons between the real and virtual pairs of matched subsets (Figure 6). We conclude that the 25.5 weeks advantage that actually GTR appears to provide over the actual (but unmatched) BX/STR controls (62 *vs* 36.5 weeks in Figure 5) can be shown by the present mathematical model to be half-accounted for by the inadvertent selection of many patients with smaller tumours to be subjected to GTR (c.f., Figure 2). If one compares the real GTR patients with their virtual controls matched by pre-operative imaging characteristics (Figure 6B), one can see that the advantage is only about 17.1 weeks, this being the difference between the median survival times of the actual 32 patients (62 weeks) and their 32 model-matched (biopsy only) virtual controls (44.9 weeks). A comparison of the two sets of virtual controls shows that those for the GTR patients survived 12.5 weeks longer than for the BX/STR patients (median=44.9 weeks in Figure 6B *vs* 32.4 weeks for Figure 6A). Clearly, the ‘better’ patients were chosen for GTR.

We believe that the present mathematical model is a powerful tool for understanding and interpreting the images displayed by different MR imaging sequences of glioblastomas since it successfully combines two of the pre-operative imaging characteristics of a glioblastoma, the ‘size’ and the ‘gradient’ of concentration of glioma cells between the ‘edges’ revealed by T1-Gd and T2 weightings of the MRI (Kelly *et al*, 1987; Dalrymple *et al*, 1994). A significant part of the power of the model is the use of the actual sizes shown in the MRIs (Figure 2). The prognostic importance of the ‘size’ has also been controversial, a few (Yamada *et al*, 1993; Xue and Albright, 1999; Jeremic *et al*, 2004) reporting it to be significant but most (Reeves and Marks, 1979; Andreou *et al*, 1983; Wood *et al*, 1988; Hammoud *et al*, 1996; Kowalczuk *et al*, 1997; Keles *et al*, 1999; Kreth *et al*, 1999; Lacroix *et al*, 2001) failing to find any relation. Xue and Albright (1999) make the point that the volume should be calculated as accurately as possible (i.e., planimetrically as the sum of the areas), as we have done, not modelled simply as spheres or ellipsoids with only the diameter(s) measured. The failures are easy to understand because of the biologic heterogeneity of all groups of patients so far studied, that is, with inherently different but unknowable rates of proliferation and invasion. These biologically fundamental rates have been subsumed within the histopathologic diagnosis (e.g., ‘generally circumscribed, slowly growing,’ ‘slow growth, and diffuse infiltration,’ ‘rapid, infiltrative growth’ etc.) (Kleihues *et al*, 2002) and have previously not been more specifically considered, much less estimated. In fact, these turn out to be the most significant of all of the factors, even better than age and KPS. It is not surprising that such previously unidentified phenotypical diversity would be impossible to control for using standard statistical analysis of groups of patients without the advantage of a mathematical model to characterise virtual controls for each individual patient, untreated or subjected to various extents of resection.

Another reason for previous failures (Reeves and Marks, 1979; Andreou *et al*, 1983; Wood *et al*, 1988; Kreth *et al*, 1993; Hammoud *et al*, 1996; Keles *et al*, 1999; Lacroix *et al*, 2001) to find an effect of ‘size’ of the tumour relates to the inherent ambiguity of any ratio; that is, the ratio *D*/*ρ* can remain the same if both *D* and *ρ* change in the same direction. Furthermore, statistical techniques generally look for linear relationships, whereas the relationship we have found for *D*/*ρ*, which we have associated with the T1-Gd and T2 radii, is highly non-linear.

These ambiguities and complexities relate to the invisible infiltration (dispersal) of glioma cells. At one theoretical extreme, for example, if there had been no dispersal, both the volume and the radius of the solid tumour could have increased exponentially with time, as postulated by Blankenberg *et al* (1995) and Haney *et al* (2001). With dispersal, however, the infiltrating cells become invisible and represent an unknowable fraction of the total, leaving the mass of those cells that remain visible (the complementary but still unknowable fraction of the total) to appear to grow quite differently: the radius actually increases linearly with time (according to Fisher's approximation) and the volume as a cubic. Neither radius nor volume increases exponentially as the classical model with a constant volume-doubling time demands. This is mathematically true even though the total number of tumour cells, both visible and invisible, may continue to increase exponentially. The measurable difference between exponential and cubic growth is relatively slight over the time intervals typically available clinically, but the consequences for longer term predictions are quite marked. The concept may be subtle but the effect is real and important. For another example, the ‘volume-doubling time’ that can be calculated at any moment in our model is not constant but decreases continuously, as actually shown in the report by Haney *et al* (2001). The concept of a volume-doubling time is meaningless for infiltrating tumours such as gliomas.

Evidence that T2 reveals not just oedema is seen in Figure 4, where the distribution of the values of *D*/*ρ* is the same whether steroid had been given or not. Of course, the short duration of administration of steroid in these patients may not have allowed any significant decrease in the T2 image (Watling *et al*, 1994). The evidence in Figure 4 suggests that the postulated difference between the hypothesised concentrations of tumour cells visualised by T1-Gd (80% of maximum) and T2 (16% of maximum) images may be very close to true, even though the reports of Kelly *et al* (1987), Kelly (1993) and Dalrymple *et al* (1994) were quantitatively far from convincing.

## CONCLUSIONS

Our mathematical model rests on only two biological factors, the net rates of proliferation (*ρ*) and diffusion (*D*), which are the ‘final common paths’ controlling cancerous growth. The comparison of the virtual results predicted by this mathematical model with the real results of 70 patients has revealed the power of the model as a ‘proof of principle’ not only in predicting durations of survival following a wide range of resections but also in accommodating the actual size of each patient's glioblastoma and the estimated ‘gradient’ of cells migrating from the ‘edge’ of the tumour. A major deficiency of the present report is the absence of a second MRI before treatment, forcing us to use the average *ρ* of 0.012 per day (Tracqui *et al*, 1995) to calculate an approximate *D* and an approximate velocity of expansion for each patient. To paraphrase Archimedes’ request for a fulcrum and a strong lever, given a second MRI without intervening treatment, the model would have allowed measurement of the actual velocity (*v*) and calculation of actual rates of dispersal (*D*) and proliferation (*ρ*) for each actual patient and the direct calculation of their matched virtual controls without all of the approximations that we had to resort to. With that information we should also be able to determine not only the degree of effectiveness of each patient's individualised therapies but also of the specificity of each therapy for affecting some step in the intricate metabolic pathways controlling *D* and/or *ρ*. Although we could not calculate *D* and *ρ* explicitly for each of the patients, the ratio *D*/*ρ* fell within the range expected for glioblastomas. We also need more data concerning the size at death, attainable by late follow-up MRI and autopsy, to define more accurately the end point, the postulated ‘fatal tumour burden.’
